# Supplementary material for: Usefulness of Measuring Serum Amyloid A Concentration in Japanese Black Cattle in Clinical Practice
Source: Vet Sci. 2023 Aug 17;10(8):528. doi: 10.3390/vetsci10080528 (PMC10459075; doi:10.3390/vetsci10080528)
Supplement: Supplementary file 1 [file vetsci-10-00528-s001.zip › vetsci-2549971-supplementary.pdf]

Table S1. Measurement parameters and method in the present study

<u>Blood cell</u><br>(Automatic blood cell calculator: SYSMEX)	Red blood cell (RBC)<br>White blood cell (WBC)<br>Lymphocyte ratio	<u>Electrolytes</u><br>(Electrolyte analyzer: Techno Medica)	Na<br>K<br>Cl
<u>Protein electrophoresis</u><br>(Fully automated capillary electrophoresis system: Sebia)	α-globulin<br>β-globulin<br>γ-globulin	<u>Thermal precipitation refractometer method</u>	Fibrinogen
<u>Biochemical analysis</u><br>(Automatic analyzer: Hitachi 7180)	Aminotransferase (AST)	Total cholesterol (T-Cho)	
	γ-glutamyltranspeptidase (GGT)	Free fatty acid (FFA)	
	Total protein (TP)	Total bilirubin (Bill)	
	Albumin (ALB)	Calcium (Ca)	
	AG ratio (AG)	Inorganic phosphorus (IP)	
	Urea nitrogen (BUN)	Magnesium (Mg)	
	Blood glucose (Glu)	Iron (Fe)	
	Serum amyloid A (SAA)	Sialic acid	
